# The quest and opportunities for air-breathing propulsion

**DOI:** 10.1038/s41526-026-00573-5

**Published:** 2026-04-01

**Authors:** Guru Sankar Duppada, Anmol Taploo, Taisen Zhuang, Jake Spinelli, Michael Keidar

**Affiliations:** George Washington University, Washington, DC USA

**Keywords:** Astronomy and planetary science, Energy science and technology, Engineering, Physics

## Abstract

This review provides a comprehensive analysis of acceleration mechanisms utilized in air-breathing electric propulsion, focusing on their fundamental principles, advantages, and the latest technological advancements. These thrusters, which utilize atmospheric gases to generate plasma and produce thrust, hold significant promise for very low Earth orbit missions due to their potential for high-efficiency propulsion. Central to their operation are the different mechanisms of acceleration. The review systematically categorizes the various acceleration mechanisms and discusses the physical principles behind these mechanisms, their integration into air-breathing propulsion architectures, and recent experimental efforts aimed at performance optimization.

## Introduction

The advancement of satellite technology and space exploration necessitates the development of innovative propulsion systems that are both sustainable and efficient^1^. Traditional chemical propulsion systems, while providing high thrust, are inherently limited by finite onboard fuel reserves which restrict mission lifespans and add significant mass and cost burdens. Another significant limitation of chemical propulsion is its relatively low specific impulse (Isp), which typically ranges from 300 to 450 s depending on the specific propellant combination and engine design. To overcome these limitations^2–4^, the focus has shifted toward electric and plasma-based propulsion systems, particularly those capable of utilizing environmental resources in situ, thereby enabling extended operational periods, and reducing overall mission costs. A tradeoff study between a satellite with an air-breathing electric propulsion (ABEP) and a traditional (Xenon-based) electric propulsion systems has been performed. It was found that ABEP equipped VLEO satellites outperform traditional EO satellites in size, weight, and power without sacrificing cost and capability^5^

Within this landscape, air-breathing electric propulsion (ABEPs) have emerged as a promising class of propulsion devices tailored for very low Earth orbit (VLEO) applications. The VLEO environment offers significant advantages^2^, including improved Earth observation resolution (up to 10 cm^6^, reduced signal latency for communication constellations, and radiation exposure levels that are 50–80% lower compared to higher orbits^7^. The reduced signal latency is a key benefit for communications, while geosynchronous orbit (GEO) satellites typically experience one-way communication delays of around 300 milliseconds due to their high altitude ( ~ 35,000 km), satellites in VLEO (very low Earth orbit, typically 200–300 km altitude) can reduce this delay by up to 200–300 milliseconds, achieving latencies of less than 10 milliseconds one-way^8^. In contrast to traditional electric propulsion systems^9^, which depend on stored propellants, ABEPs utilize the Earth’s atmospheric gases, primarily nitrogen and oxygen, as their propellant. By harnessing ionization principles and external acceleration, these thrusters can operate continuously if there is an ample supply of atmospheric gases. This capability theoretically allows for unlimited operational duration, assuming the system upholds stability and efficiency across different environmental conditions. However, sustained operation in such conditions has been constrained by atmospheric drag and the difficulty of producing sufficient thrust efficiently^10–15^.

**Fig. 1:**
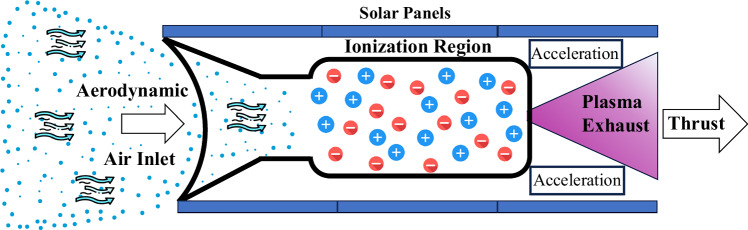
Artistic sketch of an air-breathing plasma thruster showing an aerodynamic air inlet, plasma ionization stage, and acceleration region where plasma is expelled to generate thrust.

The core operational principle of ABEPs centres on the ionization of atmospheric gases through an aerodynamic intake^13,16^, which is strategically positioned at the spacecraft’s forward section. The captured atmospheric gases^17^ are then directed into a ionization chamber, where they undergo ionization (conversion to plasma) via electron energy supplied by onboard power sources. The plasma is subjected to electromagnetic or electrostatic fields that, accelerate the ions and produce thrust as shown in Fig. 1. This approach offers several advantages for system optimization, including scalability, reduced launch costs, and diminished spacecraft mass. The effectiveness of ABEPs depends on sophisticated planning of plasma generation, ionization efficiency, and ion acceleration, which are critical for optimizing overall system performance.

To this end, the advancements in ABEPs^4,18,19^, and diverse acceleration mechanisms have been studied and implemented, each with inherent benefits and technical difficulties. A challenge in adapting existing ABEPs for efficient operation with atmospheric propellants lies in the efficient ionization of air molecules primarily nitrogen and oxygen and in ensuring the durability of thruster components exposed to reactive species. Ionizing air is more difficult than noble gases due to the higher ionization energies of N₂ and O₂, which means that these systems often require increased power input to achieve comparable ionization efficiency and thrust performance. Electrostatic acceleration methods, which typically employ grids or electrodes, can provide high specific impulse but are particularly vulnerable to rapid material degradation when subjected to high-energy oxygen ions; this results in electrode sputtering, chemical erosion, and thermal stress, ultimately limiting device lifespan^20^. Oxygen, especially in its atomic form prevalent in VLEO, is highly reactive and poses a significant threat to component longevity unless oxidation-resistant materials or protective coatings are employed. In contrast, electromagnetic acceleration techniques, including inductively coupled plasma (ICP)^21–23^, and Hall-effect thrusters operate without direct contact between electrodes and plasma, thereby limiting erosion and supporting longer lifetimes. Furthermore, component survivability and operational lifetimes remain primary considerations, especially since the power required for sufficient air ionization increases both thermal and physical stresses on materials. Magnetoplasmadynamic (MPD) ABEPs have been extensively investigated due to their high thrust density, scalability, and ability to process a wide range of propellants, including atmospheric gases. For example, NASA and ESA have developed steady-state applied-field MPD thrusters operating at power levels up to 115 kW and achieving thrust efficiencies above 40%^24,25^. These results highlight the potential of MPD thrusters for ABEP applications, although technical challenges such as electrode erosion especially when using atmospheric propellants remain a key area of research^26^.

In addition to these developments, the operation of traditional thrusters on molecular propellants such as air molecules has been explored. While conventional electric thrusters like Hall-effect thrusters and ion engines are typically optimized for noble gases, their operation with molecular propellants introduces additional challenges, including energy losses due to the ionization of molecules like N₂ and O₂. Experimental and modelling studies have shown that thrust efficiency and performance are significantly reduced when using air or nitrogen compared to xenon, primarily because a substantial portion of the input energy is consumed for ionization and non-thrust-producing excitation channels^27^. For example, tests with nitrogen in Hall thrusters have demonstrated that nitrogen yields the lowest thrust and anode thrust efficiency among tested gases, with values such as 5.7 mN and 5.4% for nitrogen compared to 12.6 mN and 26.3% for xenon^28^. These losses are attributed to additional molecular energy sinks, such as rotational and vibrational excitations, and the inherently higher ionization energy and lower ionization cross-section of molecular species. Simulation studies confirm that the poor performance of molecular propellants can be partly mitigated by increasing mass flow rates or modifying channel geometry, but efficiency remains lower than with noble gases. Reviews of alternative propellants consistently highlight that molecular propellants, including air and nitrogen, lead to degraded performance and efficiency in Hall thrusters due to these fundamental physical and kinetic limitations. As a result, ongoing research is focused on optimizing thruster design, channel configuration, and operational parameters to improve the efficiency of molecular propellant utilization, which is critical for practical and efficient ABEP systems in very low Earth orbit and planetary missions.

**Fig. 2:**
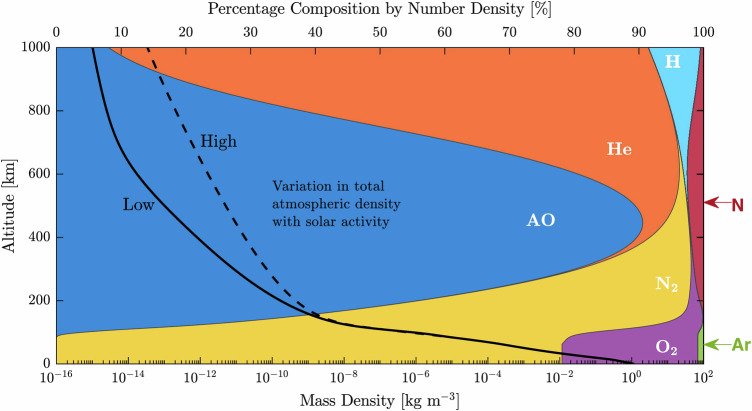
Variation of atmospheric density and composition with altitude and solar activity level in LEO, as calculated by the NRLMSISE-00 model^3^. Above 160 Km is majorly atomic oxygen.

Despite these advancements, deploying ABEPs in the environment of VLEO presents many technical challenges. Integrating ABEPs into operational systems necessitates careful consideration and ongoing research. The effectiveness of atmospheric intake in capturing sufficient particles heavily relies on several factors, including altitude, atmospheric density, spacecraft orientation, and intake design. At the lower boundary of VLEO, where atmospheric density is extremely rarefied, collection efficiency can be severely limited, potentially reducing thrust performance^16,29^. Conversely, at higher altitudes within VLEO, the diminishing atmospheric density and atmospheric variability including fluctuations in density, composition, and solar-driven disturbances as shown in Fig. 2 can impact plasma generation and the subsequent acceleration processes essential for reliable propulsion. Moreover, plasma discharge physics under these conditions is markedly different from that encountered in vacuum-based plasma thrusters^30^, which requires experimental and computational research to understand and optimize plasma behaviour, ionization efficiency, and electrode interactions in partially ionized atmospheric environments. Environmental variability also plays a critical role in system performance. Solar activity, for instance, can significantly influence atmospheric composition, density, and ionization levels, thereby affecting the consistency and predictability of propulsion efficiency^31^. Accurate modelling of these effects is required to ensure system reliability and to develop adaptive control strategies that can compensate for environmental changes.

This paper provides an overview of the various acceleration mechanisms employed in ABEP systems, highlighting their operational principles, advantages, and implementation challenges. Specifically, potential operational conditions in VLEO that are most suitable for each of these engines has been identified. Particular attention is given to electrostatic acceleration methods such as gridded ion thrusters and electromagnetic techniques including Hall-effect and MPD accelerators, with an emphasis on their feasibility for sustained operation in the very low Earth orbit (VLEO) environment.

Compared to existing reviews such as Andreussi et al. ^32^ which focus primarily on system architecture, mission design, and end-to-end technology validation of ABEP systems, this work centres on the comparative analysis of core plasma acceleration and ionization mechanisms. It examines how propulsion mode grid-based vs. Hall-type and intake strategy passive vs. compressed jointly influence key performance metrics such as thrust-to-power ratio, ionization efficiency, and erosion behaviour. Through this mechanism-level perspective, the paper aims to identify the most promising pathways toward achieving efficient, reliable, and scalable ABEP for small satellite platforms and future near-Earth space missions.

## Acceleration Mechanisms in Air-Breathing Electric Propulsion (ABEP) for VLEO

Air-breathing electric propulsion systems (ABEPs) have emerged as a promising solution for maintaining satellite altitudes in very low Earth orbit (VLEO), where atmospheric drag is significant and continuous propulsion is required. Central to the operation of ABEPs is the conversion of collected atmospheric gases into thrust, achieved through a variety of plasma acceleration mechanisms. These mechanisms are responsible for energizing ionized atmospheric particles and expelling them at high velocities, thus generating the net thrust required to offset orbital decay.

Among the available methods, electromagnetic acceleration and electrostatic acceleration are the two principal approaches adopted in ABEP thruster designs. Electromagnetic acceleration in ABEP thrusters operates primarily through the Lorentz force (J × B), which imparts momentum directly to charged particles within the plasma. The acceleration mechanism is fundamentally due to the interaction of the current density (J) within the plasma and the applied magnetic field (B). In contrast, electrostatic acceleration mechanisms rely on static or quasi-static electric fields produced by grids or electrodes to accelerate ions. Here, a series of permeable grids with precisely applied voltages create strong electric field gradients, drawing positively charged ions from a plasma source and accelerating them to high exhaust velocities. This method, seen in gridded ion thrusters and similar architectures, allows for fine control over ion energy, high propulsion efficiency, and has been demonstrated successfully in both laboratory and mission settings particularly with robust materials to mitigate erosion under continuous atmospheric intake conditions. These two acceleration approaches, electromagnetic and electrostatic form the foundation for developing reliable ABEP systems that can effectively compensate for drag and enable longstanding operations in the challenging regime of VLEO.

RF thrusters represent a newer, yet highly promising frontier in ABEP development. By RF coupling to plasma via Alfvén, ion cyclotron, or helicon waves, these methods provide contactless energy transfer and ion acceleration, significantly reducing wear and erosion associated with traditional systems. Recent experimental efforts have demonstrated the ability to excite specific plasma waves under atmospheric conditions, substantially enhancing ionization efficiency and thrust potential. Nonetheless, challenges persist in controlling wave propagation, mode coupling, and mitigating undesired wave modes across varying atmospheric pressures and densities. Progress in real-time impedance matching and adaptive mode control systems could further unlock their potential, leading to longer operating life and scalable configurations. Electrothermal acceleration, another relevant mechanism, involves resistive heating, microwave, or plasma arc methods to rapidly elevate gas temperature and pressure, producing thrust via expansion through nozzles. While primarily used in thermal propulsion, electrothermal principles can also facilitate ion acceleration through thermal ionization of atmospheric gases. Its main drawback lies in lower efficiency and high energy requirements, resulting in thermal management challenges and limited practicality for sustained, high-thrust operations in VLEO environments.

### MPD acceleration

**Fig. 3:**
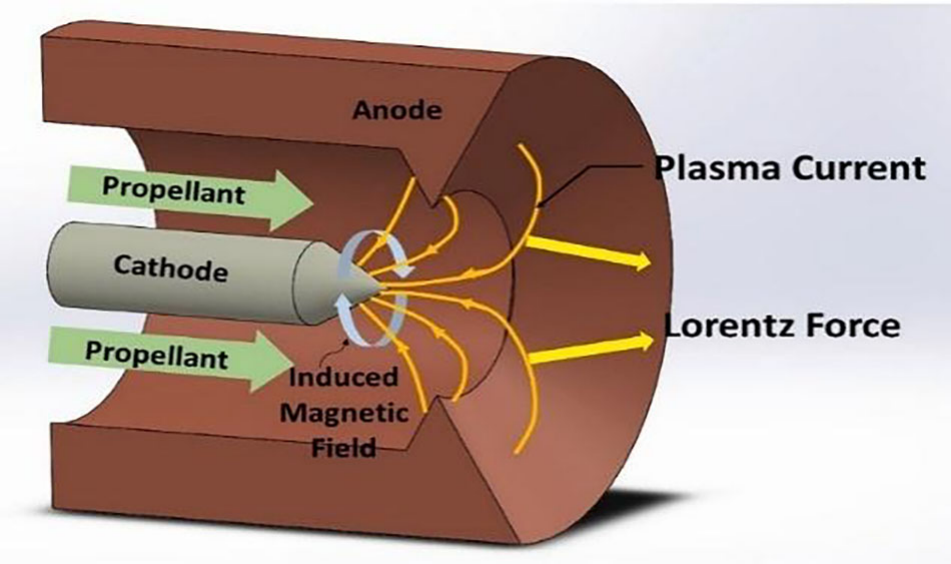
General overview and cross-sectional view of MPD thruster^35^.

The MPD acceleration mechanism operates by capturing atmospheric gases, such as oxygen and nitrogen, and ionizing them to form a plasma within the thruster. In the MPD thruster as shown in Fig 3, two key electromagnetic fields are established: an electric field, generated by the applied voltage between electrodes, and a magnetic field, which can be created either by the current itself (self-field MPD) or by external coils or magnets (applied-field MPD)^33^. In the self-field mode, the current passing through the plasma induces an azimuthal magnetic field encircling the cathode via Ampere’s Law, while the applied-field approach introduces a strong axial magnetic field, enabling more robust and controlled operation, especially at lower power levels. Regardless of the magnetic field source, the central physical process is the creation of a Lorentz force where the electric current density flowing radially through the ionized plasma interacts with the magnetic field, resulting in a force that accelerates the plasma axially out the exhaust at very high velocities typically tens of kilometres per second^34^.

This electromagnetic acceleration mechanism provides high thrust density and excellent scalability, allowing MPD thrusters to generate the continuous, dense thrust required to counteract atmospheric drag in VLEO, with experiments demonstrating thrust levels from several millinewtons up to tens of newtons at very high input powers (100 kW and above). The capability to operate with a variety of propellants including in-situ atmospheric gases makes MPD thrusters ideal for air-breathing propulsion, enabling satellites or spacecraft to use the ambient atmosphere as a virtually unlimited propellant supply, thus dramatically extending mission duration and reducing mass constraints^35^.

MPD thruster designs come with their own engineering challenges. The highly reactive atomic oxygen found in VLEO can erode electrodes and structural components rapidly, demanding advanced materials and design strategies to ensure operational longevity. Applied-field configurations offer higher efficiency (up to 60%) and improved control but at the cost of added complexity and often heavier magnetic hardware. Ongoing research focuses on optimizing intake efficiency, ionization processes, and plasma stability to maximize performance while minimizing component wear.

### Pulsed Inductive Thruster (PIT) Acceleration

**Fig. 4:**
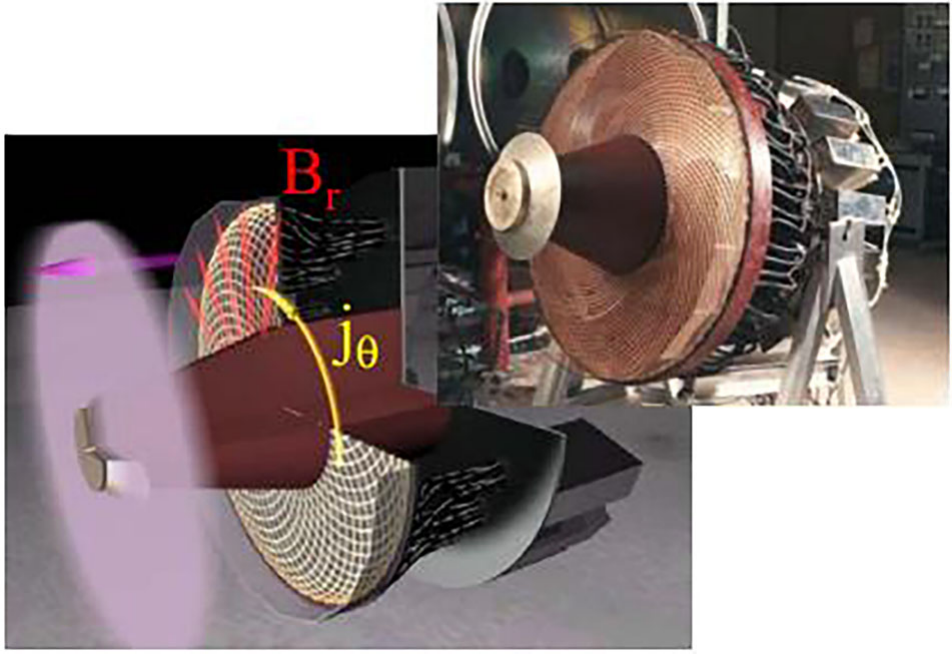
The PIT and a schematic of the relative acceleration process due to the dominant interaction of the applied radial field and the induced azimuthal current^63^.

The operational cycle of a PIT begins with the injection of a small puff of neutral gas such as argon, xenon, ammonia, or even atmospheric air into the region above a flat or spiral induction coil, as shown in Fig. 4. When a high-voltage (∼10–50 kV), high-current pulse is delivered to the coil, it generates a rapidly changing magnetic field. According to Faraday’s law of induction *((∇ × E* = *−* $\frac{\partial B}{\partial t}$*)*, this time-varying magnetic field induces strong azimuthal (circular) eddy currents in the neutral gas. The energy imparted by these currents is sufficient to ionize the gas, transforming it into a plasma in a matter of microseconds. Once ionized, the plasma interacts with the magnetic field produced by the coil. The key to PIT operation is the Lorentz force, which arises from the interaction between the induced plasma currents and the magnetic field. This force acts perpendicular to both the current and the magnetic field, accelerating the plasma axially away from the coil and thereby generating thrust. Because the entire process is electrodeless meaning there are no physical electrodes in contact with the plasma, PITs avoid the electrode erosion and degradation that limit the lifespan and reliability of many other electric propulsion systems, such as Hall effect thrusters or MPD thrusters.

PITs offer several notable advantages. Their electrodeless design enables compatibility with a wide variety of propellants, including reactive or corrosive gases like atmospheric air or carbon dioxide, which would quickly degrade conventional thruster electrodes. This makes PITs particularly well-suited for air-breathing electric propulsion in VLEO, where they can utilize the residual atmospheric gases as propellants, enabling extended mission lifetimes at altitudes where drag is significant. Additionally, PITs can be scaled for different power levels and mission requirements, from small satellites to large spacecraft.

Despite these advantages, PITs face several technical challenges. The requirement for high-voltage, high-current pulse power supplies necessitates robust and efficient energy storage and switching systems, such as large capacitors and fast solid-state switches. Achieving optimal coupling between the ionization and acceleration phases demands precise timing and control of the energy pulse. Furthermore, only a fraction of the input electrical energy is converted into kinetic energy of the exhaust plasma, with typical thrust efficiencies ranging from 30% to 50%. Research is ongoing to improve this energy coupling and to address issues such as plasma instabilities and current sheet detachment, which can reduce performance.

Recent advancements include simulation and laboratory studies exploring how air plasma chemistry influences current sheet formation and thruster performance. Several research initiatives^36^ are actively investigating air-breathing inductive plasma thrusters, aiming to understand and optimize their operation with atmospheric gases like N₂, O₂, and CO₂^37^. Notably, experimental prototypes and modelling efforts have demonstrated that with suitable plasma chemistry and optimized pulse timing, inductive thrusters can operate efficiently on air-like mixtures, showing performance trends comparable to those with noble gases, but at different power levels. These developments support the feasibility of using PITs for ABEP applications. Moreover, theoretical work and early experiments have explored the potential for PITs to utilize planetary atmospheres such as Mars’ CO₂-rich environment highlighting their suitability for in-situ resource utilization and refuelling on planetary surfaces. These advancements collectively indicate that, although experimental demonstrations are still progressing, the active research landscape strongly supports the potential of inductive plasma thrusters for air-breathing operation in VLEO environments.

### Hall effect thrusters

While the primary ion acceleration mechanism is electrostatic, the applied magnetic field plays a crucial role in confining electrons and maintaining the electric field within the quasi-neutral plasma. The interplay between electric and magnetic fields is fundamental to HET operation, as the magnetic field not only enhances electron confinement but also facilitates the establishment of the potential drop necessary for efficient ion acceleration.

**Fig. 5:**
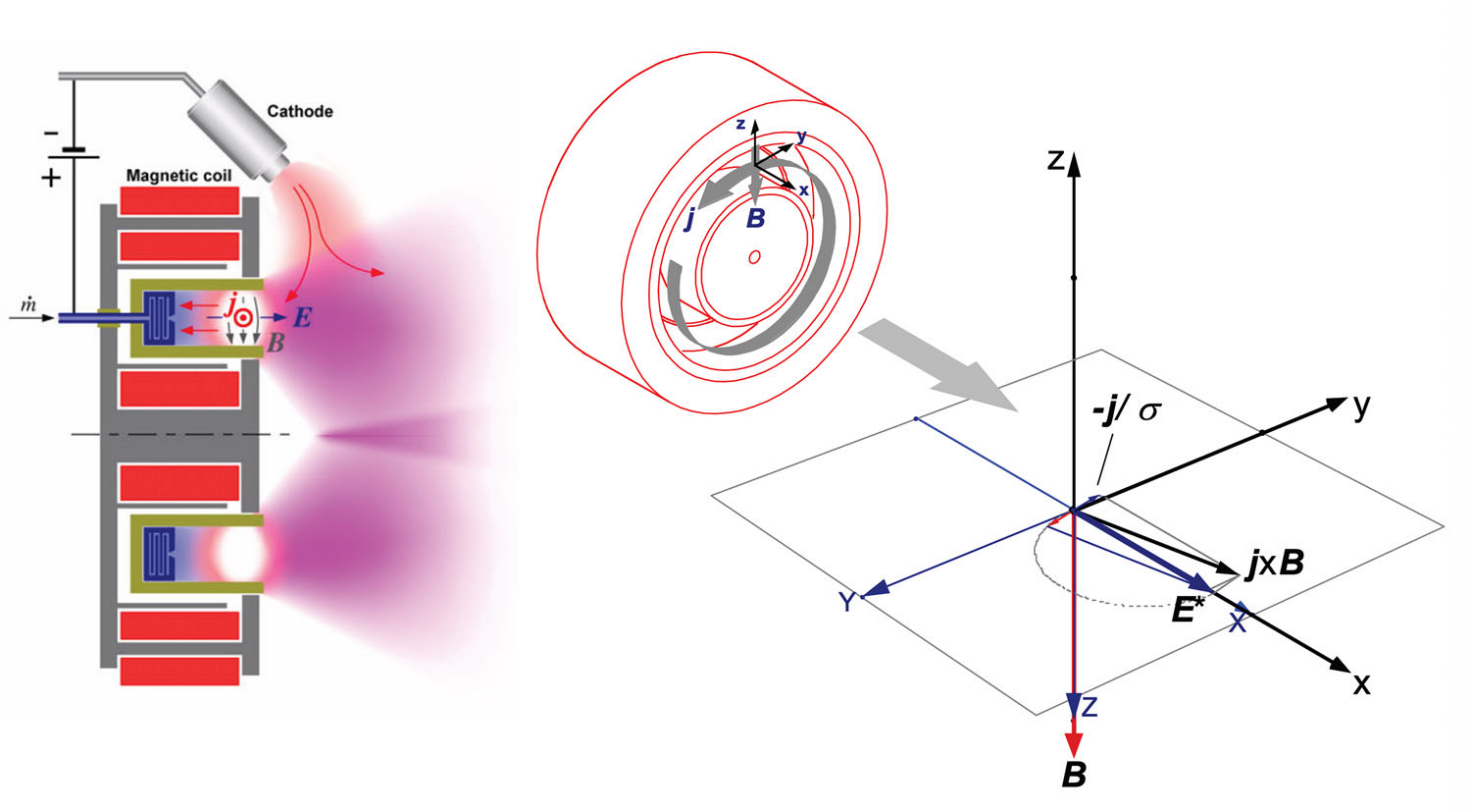
Schematic of Hall effect thruster with the acceleration mechanism^64^.

In the context of air-breathing Hall Effect Thrusters (ABHETs), these electromagnetic effects become even more pronounced as shown in Fig. 5. Air-breathing variants integrate atmospheric intake and ionization mechanisms ahead of the acceleration channel, allowing the thruster to utilize residual atmospheric gases as propellants at VLEO altitudes. The presence of a more complex and variable propellant mixture, such as atomic oxygen and nitrogen, can amplify the role of electromagnetic interactions in stabilizing the plasma and optimizing ion acceleration. Additionally, the design of the magnetic field topology becomes critical for ensuring efficient ionization, confinement, and acceleration of the diverse atmospheric species encountered in VLEO^38,39^.

### Grid-Based Ion Acceleration (Gridded Ion Thrusters)

**Fig. 6:**
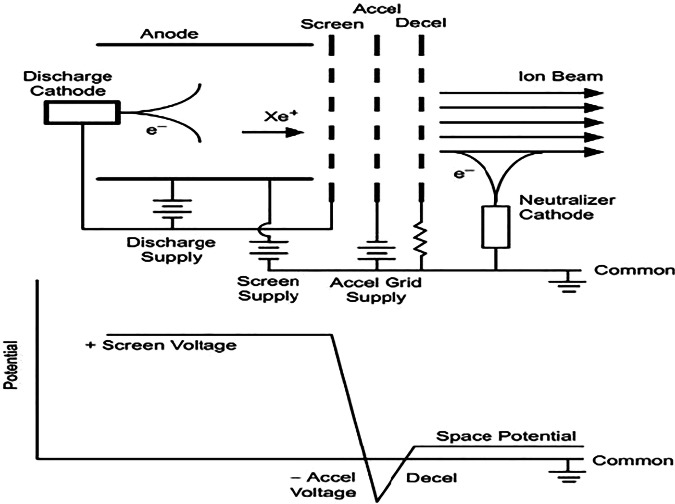
Electrical schematic of a Gridded ion thruster without the cathode heater and keeper supplies^65^.

Grid-based ion acceleration is widely employed in ion thrusters. In this approach, ions generated within a plasma are extracted and accelerated through carefully spaced and electrically biased grids. The typical grid structure as in Fig. 6 consists of an ion extraction grid, positively biased to draw ions from the plasma sheath, followed by one or more accelerator grids that impose a strong electric field across the gap. This field accelerates the ions to high velocities, producing a narrow, collimated ion beam. The grids are meticulously engineered to ensure uniform electric fields, minimize beam divergence, and reduce the risk of electrical breakdown or arcing between electrodes. The operational sequence begins with plasma generation, commonly achieved by ionizing a noble gas such as xenon using electron bombardment or radio-frequency (RF) discharges. The resulting positively charged ions are extracted through the first grid and then accelerated by the potential difference established between subsequent grids. After acceleration, a neutralizer typically a hollow cathode or field emission device emits electrons into the ion beam to neutralize its charge, preventing the buildup of space charge that would otherwise cause beam divergence and spacecraft charging.

This electrostatic acceleration technique imparts substantial kinetic energy to the ions, enabling the generation of high-velocity exhaust beams and, consequently, high specific impulse (Isp) values. Grid-based ion thrusters^15^ can achieve specific impulses in the range of 2,000–10,000 s, far surpassing the performance of chemical propulsion systems. The precise control over grid voltages allows for fine modulation of thrust magnitude and direction, making these thrusters highly suitable for deep-space missions, station-keeping, and fine attitude adjustments. Missions employing grid-based ion acceleration include NASA’s Deep Space 1 and Dawn spacecraft, both of which demonstrated the efficiency and reliability of ion propulsion for long-duration interplanetary travel^40^. However, grid-based ion acceleration is not without challenges. One of the primary limitations is grid erosion, caused by direct ion impact or sputtering, which can degrade performance and ultimately limit thruster lifetime. This is particularly problematic in air-breathing electric propulsion concepts operating in very low Earth orbit (VLEO), where reactive atmospheric species such as atomic oxygen can severely degrade the exposed material. Additionally, the need for high-voltage, high-precision power supplies and the risk of voltage breakdown between closely spaced grids require robust engineering solutions. Advances in grid materials, such as carbon-carbon composites and molybdenum alloys, as well as improved grid geometries, are ongoing to address these issues^41,42^.Furthermore, the same gridded acceleration technique can, in principle, be adapted to air-breathing ion engines, where ionization and acceleration are performed using ambient atmospheric gases. Although originally developed for operation in vacuum with noble gases like xenon, recent research efforts have explored the feasibility of adapting gridded ion thruster architectures to operate on molecular species such as N₂ and O₂, highlighting both the promise and the unique challenges of implementing such systems in the VLEO environment^43^.

## A case for Air-breathing Hypersonic Electric Propulsion

Most approaches focus on achieving air compression by forming inlet collimation. However, for a typical thruster operation a very large air compression is necessary. For instant, Diamant^5^ proposed a two-stage cylindrical Hall-effect thruster, combining an ECR ionization stage with a cylindrical Hall thruster architecture, predicting operation at ~220 km altitude if ambient air is passively compressed by roughly ×500. However, such passive compression (using diffusers or collimators) is likely impractical and could introduce excessive drag. Indeed, SITAEL’s RAM‑EP testing demonstrated measured thrust of 6 ± 1mN while the system drag reached 26 ± 1mN, resulting in a net negative thrust during ground-based simulations of VLEO air intake^44^.

**Fig. 7:**
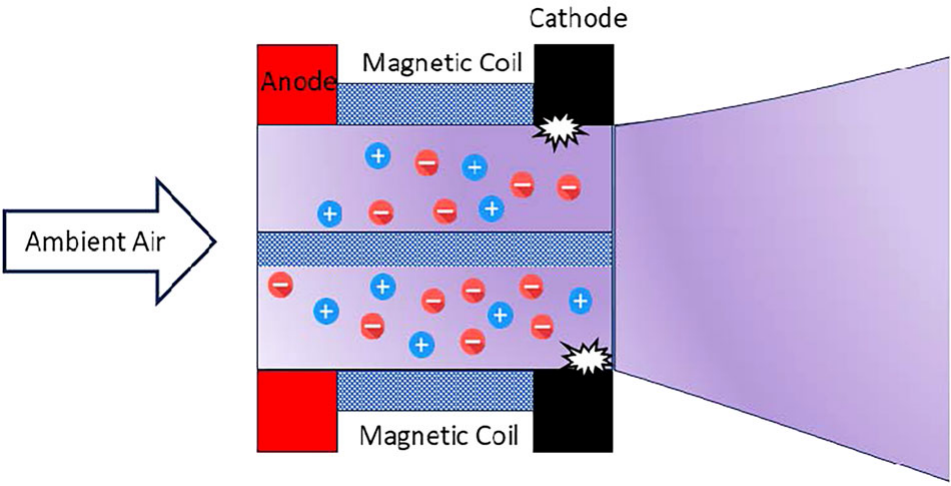
Schematic of air-breathing Hall thruster.

In this Section we consider a possibility of operating air-breathing electric thruster without air collimation and compression. It has been shown that conceptually, such a thruster can indeed work effectively at the orbits of about 90–95 km, producing significant thrust in the range of 9.1–22 N for considered conditions^45^. Schematically, this thruster is shown in Fig. 7. Modelling results show that as altitude increases, the required power to sustain full ionization and maintain thrust output increases, primarily due to the decreasing atmospheric density at higher altitudes. Conversely, at lower altitudes, the higher atmospheric density results in increased collisions, which may affect plasma uniformity and ion acceleration efficiency. Thus, it can be concluded that 90–95 km orbit range is optimal for the airbreathing Hall thruster operating in the full ionization mode without compression. This approach to EP thruster without compressor was recently proposed but it requires formation of almost fully ionized air. Thus, one of the highly challenging problems is to discover conditions for strong ionization. In turn, strong ionization will lead to high current and consequently high power required ( < 100 kW).

Recent more detailed studies suggested that air-breathing thruster can operate at altitudes of 80-100 km without the need for air compression, utilizing a scramjet-like configuration^46^. To that end, efficient air ionization approach based on electron beam ionization has been developed and implemented^47^. In addition, it has been discovered that air-breathing thruster operation at these altitudes provides conditions for self-neutralization^48^.The ultimate requirement for electric propulsion systems, especially considering limited onboard resources, is to attain a high thrust-to-power (T/P) ratio^49^, which can be expressed as:

1 $$\frac{T}{P}\,\approx \frac{2}{{V}_{{ex}}-{Vo}}$$

where ${V}_{{ex}}$ is the exhaust velocity and Vo is the initial velocity of the air entering the inlet, i.e. flight speed. It should be noted that Eq. (1) is not an exact equality, but a first-order approximation derived under ideal assumptions, such as steady flow, negligible losses, and efficient momentum transfer, as discussed by Goebel and Katz (2008). Theoretically, if the velocity increase imparted by the thruster is small, the T/P ratio can be maximized. This highlights the importance of the scramjet-like configuration in optimizing performance by maintaining a high T/P ratio. However, this leads to thrust decrease imposing an important trade off.

To sustain high thrust levels, it becomes necessary to utilize a larger thruster cross-sectional area to ensure a sufficient mass flow rate. This highlights the importance of optimizing the balance between minimizing energy losses during ionization and maintaining a high mass flow rate, which is crucial for developing effective high-altitude air-breathing electric propulsion systems. However, what clear is that high T/P can be achieved by not slowing down the air flow. What studies showed is that practically achievable altitudes are in the range of 80-100 km. An additional benefit is that it was recently discovered that at these altitudes, the air density is such that self-neutralization becomes possible^48^.

Taploo et al. designed an air-breathing MPD thruster^50^ based on the hypersonic electric propulsion concept described above. In this configuration, the incoming hypersonic flow serves as the propellant, which is ionized and subsequently accelerated through Lorentz force. The proposed air-breathing MPD concept demonstrated high thrust-to-power ratios while producing nominal thrust levels suitable for operation in the 65–120 km altitude range, where atmospheric densities are significantly higher. These results highlight the potential of MPD-based hypersonic electric propulsion for VLEO missions.

**Fig. 8:**
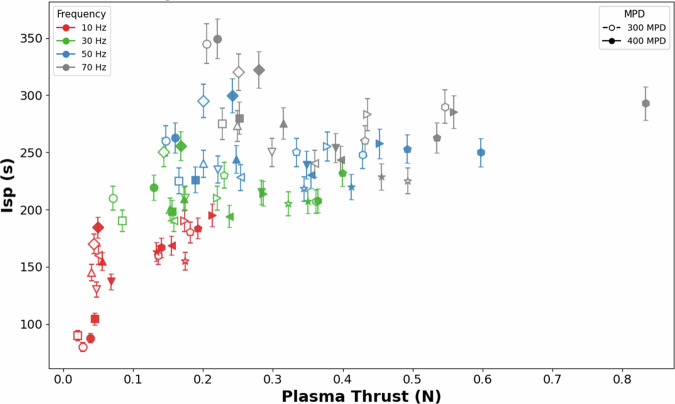
Specific Impulse as a function of thrust due to plasma. Isp is calculated based on experimental data presented in Ref. ^50^.

Figure 8 shows the relationship between Isp and plasma-generated thrust for an air-breathing MPD thruster operating under hypersonic electric propulsion conditions^50^. The results show a systematic increase in Isp with increasing plasma thrust, reflecting more effective acceleration as discharge energy and current density increase. Variations in acceleration voltage and pulse repetition rate shift the operating points along this Isp–thrust envelope, with higher repetition rates generally enabling higher Isp at a given thrust level. In this altitude range of 65-120 km, the availability of dense incoming flow allows the MPD thruster to operate in a regime where both thrust and Isp can be increased simultaneously, as reflected by the upper envelope of the data in Fig. 8. This behaviour supports the suitability of air-breathing MPD concepts for hypersonic propulsion and drag-compensation missions at extremely low altitudes.

## Summary of available thruster approaches for VLEO

The development of ABEP systems for VLEO applications depends upon the careful selection and optimization of acceleration mechanisms to achieve an optimal balance among performance, durability, and operational complexity. A comparative overview of ion propulsion techniques such as electrostatic, electromagnetic, and electrothermal highlighting the unique strengths and trade-offs associated with each approach is summarized in Table 1. Most ABEP systems described in Table 1 incorporate a “scoop” or intake mechanism at the front of the spacecraft, which is essential for collecting ambient atmospheric particles to be used as propellant. This intake becomes especially necessary at higher altitudes within the VLEO regime, where the atmosphere becomes increasingly rarefied. In these conditions, the efficiency of air collection and delivery to the propulsion system is a defining factor in overall system performance and mission feasibility. By integrating a properly designed scoop, ABEP systems can efficiently utilize the residual atmospheric gases available in VLEO, thereby compensating continuous drag and extended satellite lifetimes without reliance on onboard propellant.

**Table 1:** Estimated or typical performance characteristic values based on experimental data and literature reviews

#	1	2	3	4	5	6	7	8
**Thruster**	New Orbit RF ABEP	RF Helicon IPT	AETHER HT5k	RAM-HET	PPS1350-TSD	RIT-10	TAMU Pulsed Plasma MPD Thruster	Air-breathing MPD thruster
**Mechanism**	Electrodeless RF / hybrid	Electrodeless RF inductive	Hall effect thruster	Two-stage Hall thruster	Hall effect thruster	Gridded electrostatic ion	Electro magnetic	Magnetoplasmadynamic accelerator
**Thrust (mN)**	15.1	single- to low-double-digit mN	64	6 ± 1	~21 mN	5.25 (N₂); ~6 (O₂)	~19 at 100 Hz	>1600
**T/P (mN/KW)**	13.4	~5–15	26.7	20	Not Stated	11.7 (N₂); 13.3 (O₂)	~21–30	5000
**I**_**sp**_ **(s)**	~800–1200	~500–1000	~1500–2000	~1500–2000	~1500–2000	~3000	~3500	600 - 6000
**Efficiency (%)**	~25–35%	~30–40%	~50%	Not stated	~50%	~20–30%	~40%	--
**Altitude (mapped/test)**	Lab, VLEO flow	Model and low-power lab, 100–250 km	Lab, ~200 km	Lab, ~200 km	Lab, ~200 km	Lab, ~200 km	Not Stated	65–120 km
**Scoop**	Yes	Yes	Yes	Yes	Yes	Yes	Intake assumed	Yes
**Neutralizer**	Yes	Maybe	Yes	Yes	Yes	Yes	No	No
**Scoop type**	PFG / lab flow	Honeycomb / duct	PFG/lab supply	Split-ring + conical duct	PFG / lab supply	PFG / lab supply	Not Stated	
**Advantages**	Recent real T/P data	Electrode-less; good for reactive species	Higher T/P; practical ABEP	Integrated scoop–thruster	Better erosion resistance	High I_sp_; mature	High T/P, Adjustable Pulse rate	High Thrust and T/P occupying entire VLEO
**Disadvantages**	Requires system integration	Less mature; high complexity	not flight-scale	net thrust low	Thrust lower on pure air; intake needed	Grid erosion; sensitive to molecules	Pulsed system integration complexity	Consumes KW of power
References	^50^	^56^	^57^	^58^	^59^	^60^	^61^	^62^

Some values may vary depending on specific configurations, propellants, and operational regimes The altitudes mentioned in the table were directly taken from the referenced studies. It should be noted that the reported thrust and thrust-to-power values for Aether HT5k, PPS1350, and RIT 10 thrusters correspond to ideal laboratory operating conditions, in which neutral particles are supplied using mass flow controllers or particle flow generators at densities several orders of magnitude higher than those available in VLEO. These results should therefore be interpreted as demonstrations of the acceleration stage rather than complete air-breathing propulsion performance.

ABEP in refs. ^2–5^. is primarily defined as an enabling technology for sustained operations in VLEO, where atmospheric drag becomes the primary concern and continuous thrust is required to counteract orbital decay. Target altitudes typically below 250 km impose mission requirements that differ fundamentally from those of conventional electric propulsion, as propulsion performance in VLEO is constrained by the availability of ambient neutral particles rather than by stored propellant. Within this framework, the primary mission requirement is the ability to generate sufficient time-averaged thrust to balance aerodynamic drag under highly fluctuating conditions. The incoming neutral particle density in VLEO is both low and strongly dependent on altitude, solar activity, and orbital geometry, leading to fluctuations in the effective mass flow rate available to the propulsion system. Thus, propulsion concepts intended for VLEO must therefore tolerate non-uniform, unregulated inflow while maintaining stable discharge operation.

**Fig. 9:**
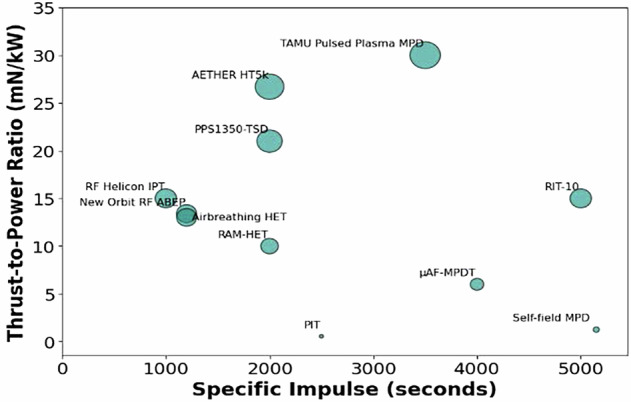
Comparison of Thrust to power vs specific impulse for various acceleration mechanisms discussed above.^38,45,64,66–69^.

From a system perspective, thrust-to-power ratio is a key merit for drag-compensation missions, as available onboard power is limited and thrust must be delivered efficiently over long durations. In contrast to high-power electric propulsion systems designed for orbit raising or deep-space manoeuvres, ABEP systems in VLEO are required to operate continuously at modest thrust levels with high reliability and minimal operational complexity. Furthermore, it is important to emphasize on system simplicity and scalability for small satellite platforms, where mass, volume, and power margins are particularly constrained. Propulsion concepts that minimize the need for sophisticated magnetic field configurations, precise propellant flow control, or high-voltage ignition schemes are therefore attractive from a mission integration standpoint. In this context, the results presented here are discussed in terms of their relevance to sustained VLEO drag-compensation capability, rather than absolute performance metrics obtained under idealized laboratory conditions. The figure comparing thrust to power, and specific impulse for the acceleration mechanisms discussed above is shown in Fig. 9.

Electrostatic and electromagnetic systems occupy distinct regions on the T/P vs Isp plane. While electrostatic thrusters achieve high specific impulse at modest T/P ratios, electromagnetic systems like Hall thrusters offer a more balanced trade-off, especially in the medium power regime ( ~ 1–5 kW), making them suitable for scalable VLEO platforms. Electrostatic acceleration remains a foundational technique in plasma propulsion, leveraging high-voltage grids or electrodes to produce strong electric fields that efficiently accelerate ions outward. Its main advantage lies in the precise control over ion trajectories and velocities, enabling fine-tuned thrust vectoring and optimization of efficiency. Nevertheless, electrode erosion presents significant durability challenges, especially under continuous operation in reactive atmospheric conditions typical of VLEO, where atomic oxygen and other species accelerate material degradation. Although advancements in dielectric ceramics and composite materials have extended component lifespans marginally, erosion remains a critical limiting factor. Scaling electrostatic systems to higher power levels or adapting them to variable atmospheric pressures introduces additional design complexities that threaten reliability and long-term operation. The erosion-related characteristics and mitigation strategies across representative ABEP acceleration mechanisms are summarized in Table 2. Contactless designs reduce material degradation, whereas electrode-based systems require advanced material and structural solutions for operation in reactive VLEO environments

**Table 2:** Plasma–surface erosion characteristics and mitigation strategies for ABEP propulsion mechanisms

Propulsion Mechanism	Plasma Contact Surface	Erosion Level	Typical Materials	Mitigation Strategies	References
Grid Ion Thruster	Extraction grids	High	Mo, C/C composite	ALD coatings, grid geometry, magnetic shielding	^20,42,62^
Hall Effect Thruster	Channel walls	Medium	BN-SiO₂ ceramics	Magnetic shielding, segmented channel, ceramic liner	^27^
MPD Thruster	Anode, cathode	Very High	Graphite, Molybdenum	Pulsed operation, cooling loops, material hardening	^24,26^
Helicon Thruster	Dielectric tube (minimal)	Low	Quartz, alumina	Contactless RF design, impedance matching	^39^

In contrast, electromagnetic acceleration mechanisms including Hall-effect thrusters and MPD thrusters offer distinct advantages owing to their electrode designs, where electrodes do not actively contribute propellant mass to plasma generation. These systems utilize magnetic fields to induce currents within the plasma, generating Lorentz forces that propel ions without the need for physical grids. Such designs improve reliability and operational lifespan, making them attractive for VLEO applications. However, Hall-effect thrusters and ion thrusters generally require carefully tailored magnetic field configurations to ensure effective electron confinement and efficient ionization, which introduces additional design and integration complexity. These requirements typically necessitate multiple magnetic coils, precise magnetic field shaping, and dedicated power and control electronics, thereby increasing overall system complexity particularly for small-satellite platforms^51^. In contrast, propulsion concepts that rely on electrodeless or weakly magnetized discharges can reduce magnetic subsystem requirements, potentially simplifying system integration for VLEO applications.

The Very Low Earth Orbit (VLEO) environment can be broadly divided into two key regions, each significantly influencing the design and operation of Air-Breathing Electric Propulsion (ABEP) systems. At lower altitudes, approximately between 0 and 160 km, the atmosphere’s composition closely resembles ground-level air, facilitating more conventional air-breathing propulsion processes. In this regime, the focus is on efficiently capturing atmospheric gases, where higher capture efficiency and reduced surface erosion are advantageous. Power management is critical here, as the energy required to sustain ionization and compensate for atmospheric drag must be carefully balanced to ensure effective propulsion. In contrast, at altitudes ranging from 160 to 300 km, the environment becomes rich in atomic and molecular oxygen, presenting a set of unique challenges. Atomic oxygen is highly reactive and difficult to ionize due to its high ionization potential, demanding substantial power input for plasma generation and acceleration. This high energy requirement impacts the overall efficiency, as it results in lower mass utilization and capture efficiency. Moreover, prolonged exposure to atomic oxygen leads to significant surface erosion of thruster components such as intake surfaces and electrodes, threatening their durability and long-term reliability. These erosive effects could shorten mission lifetimes. At even higher altitudes, approximately between 300 and 450 km, the atmosphere becomes increasingly rarefied, and the primary challenge shifts to the collection of atmospheric gases via scoops or intake mechanisms. The low density severely limits the amount of usable propellant, making efficient collection and acceleration of scarce particles vital for propulsion. Throughout these regimes, power generation and consumption remain critical considerations. At lower altitudes, energy must support ionization and compensate for drag, while at higher altitudes, it focuses on maximizing the intake and acceleration of limited atmospheric particles^52^.

**Fig. 10:**
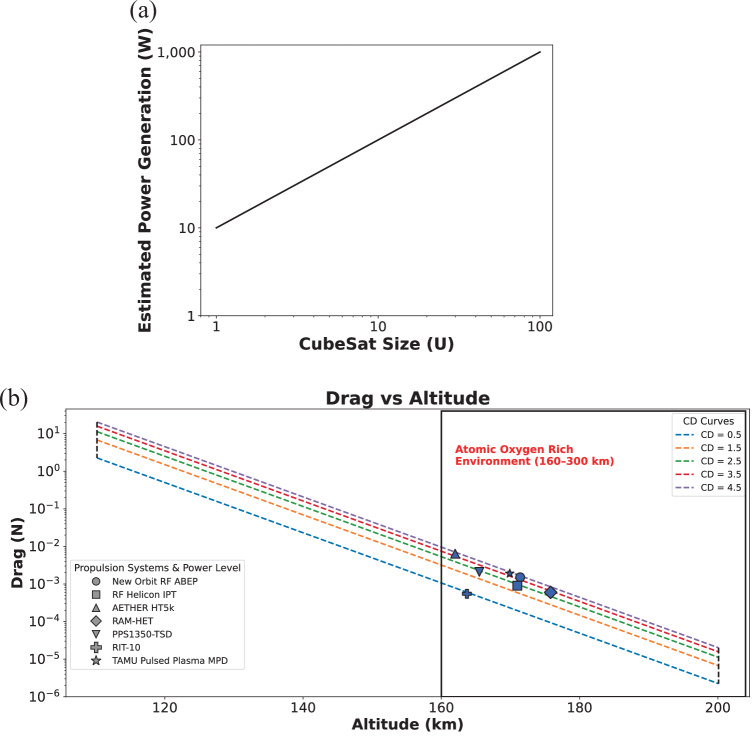
**a** Estimated Power Generation for CubeSats with Deployable Solar Arrays (1U–100 U), (**b**) Thrust required by various propulsion systems to maintain a satellite in thrust-drag equilibrium at different altitudes in VLEO. The plot includes several propulsion systems, such as the New Orbit RF ABEP^62^, TAMU Pulsed Plasma MPD Thruster^56^, RIT-10^57^, PPS1350-TSD^58^, RAM-HET^59^, AETHER HT5k^60^, RF Helicon IPT^61^. The thrust values represent the force necessary to counteract atmospheric drag and sustain orbit. All parameters consider air as the propellant and are calculated with the use of a scoop. For these calculations, the scoop size is limited to the satellite’s dimensions.

The power generation estimates for CubeSats as in Fig. 10(a) are based on a few simplifying assumptions to make the scaling consistent and practical. First, it assumes that each CubeSat has deployable solar panels that maximize exposure to sunlight, not just fixed body-mounted panels. The effective power density used is approximately 410 W/m², which is a realistic value for high-efficiency triple-junction solar cells operating in space^53,54^. Another key assumption is that available solar array area scales linearly with CubeSat size, which simplifies the complex reality of mechanical design, deployment constraints, and orientation dynamics. Essentially, it assumes that a larger CubeSat can deploy proportionally larger solar panels, even though in practice, geometry, attitude control, and thermal limitations might restrict that. The power values were scaled from a baseline of ~8 W for a 1U CubeSat, extrapolating linearly to ~820 W for a 100U, in line with performance trends from real missions^54^. This makes the data useful for high-level comparisons, but actual designs will vary depending on mission architecture and system efficiency. It should be noted that the current analysis is an idealized first-order estimate and does not include power losses in the propulsion system or spacecraft bus. Including these losses would increase the required input power and reduce the achievable thrust-to-power performance. However, since the same ideal assumptions are applied consistently across all the cases, the trends and conclusions remain unchanged, particularly the dependence on altitude and drag coefficient.

Figure 10b presents a comparative analysis of the thrust required by different propulsion systems to counteract atmospheric drag and maintain orbital stability at various altitudes. The study assumes a steady-state equilibrium, where the required thrust matches the atmospheric drag force that varies with altitude, primarily due to changes in atmospheric density. At lower altitudes, such as around 90 km, the atmospheric density is higher, resulting in greater drag. Consequently, maintaining a stable orbit at these altitudes demands significantly higher thrust from the propulsion system. The analysis also incorporates the influence of the satellite’s shape and material properties, represented by the drag coefficient (Cd). Satellites with higher Cd values experience greater drag at a given altitude, which in turn increases the thrust required to preserve orbital stability. The plot in Fig. 10b includes multiple Cd curves, illustrating how variations in satellite design impact the drag force and the corresponding thrust requirements across different altitudes. Cd values were chosen based on the extreme ranges for small satellites operating in VLEO. Reported drag coefficients typically fall in the range 1–3. For Small Satellites specifically, simulations show Cd ≈1.24 at less than 150 km in free molecular flow, with broader literature values of 2.0–2.5 for nominal cases; extremes near 0.5 or 4.5 are possible but rare, driven by optimized low-drag surfaces or high-drag configurations like deployable. For each propulsion system, the operational altitudes are indicated, with thrust values aligned to the drag calculated for their respective Cd curves. These thrust values are extracted directly from referenced studies. An important consideration highlighted by Table 1 is the relationship between thrust and power for the propulsion systems. Typically, these systems generate tens of millinewtons (mN) of thrust per kilowatt (kW) of supplied power. However, in practical spacecraft operations, dedicating kilowatts of electrical power solely to the thruster is highly challenging, given competing system demands and limitations on available onboard energy. Therefore, it is essential to account for the realistic power allocation and efficiency when evaluating the feasibility of sustaining orbital altitude with these propulsion systems.

The drag force was calculated using the standard aerodynamic drag equation

2 $${\rm{D}}=\frac{1}{2}\,\rho {V}^{2}A{C}_{d}$$

Where, D = Drag, *ρ* (atmospheric mass density) was obtained from ref. ^55^, *v* = 7.8 km/s, *A* = depends on power requirement and mission profile (the satellite’s cross-sectional area), ${C}_{d}$ is the drag coefficient, which varies depending on the satellite shape and material properties. Using these parameters, the drag force was computed for each altitude using Eq. (2), thus determining the thrust needed to compensate for atmospheric drag and maintain orbit. At 100 W (analogous to a 12U CubeSat with ideal conditions), the thrust capacity is scaled accordingly; at 500 W ( ≈ 60U), and at 1 kW ( ≈ 120 U), which corresponds to a satellite with a cross-sectional area of approximately 1 m² for 1 KW systems, 0.5 m² for 500 W systems and 0.1 m² for 100 W systems, suitable for generating power from solar panels at these altitudes. This means that the estimated drag assumes an area of 1 m², representing the satellite’s cross-sectional area. Therefore, the thrust values are directly proportional to the power input, and the figure serves as a comparative tool to visualize the thrust requirements of different propulsion technologies across various operational altitudes and drag profiles.

In terms of performance, electrostatic grid-based ion thrusters achieve high specific impulses ranging from 3,000 to 10,000 s with thrust levels between 10 and 100 mN and power requirements from 0.1 to 70 kW. These systems excel in precision and efficiency but are hindered by electrode erosion. Field emission electric propulsion (FEEP), offering lower thrusts (0.01–1 mN) and specific impulses similar to electrostatic systems, consumes less power (30–60 kW), making it suitable for small satellite missions.

## Conclusion

This review highlights the complexity of acceleration methods used for ABEP systems in VLEO orbits. As these thrusters rely on in-situ atmospheric gases to propel spacecraft, no single acceleration method can simultaneously satisfy all performance and operational requirements across the wide range of altitudes, flow conditions, and power constraints. Each acceleration approach therefore presents a distinct balance of advantages and limitations. The comparative analyses show that propulsion concepts requiring high exhaust velocity or significant flow compression rapidly become power-limited, particularly for small spacecraft. In contrast, concepts that minimize the velocity increment imparted to the incoming flow achieve higher thrust-to-power ratios and are better suited for continuous drag compensation, albeit at the expense of reduced absolute thrust. The drag analysis further demonstrates that ABEP feasibility is highly sensitive to altitude, spacecraft geometry, and surface properties, underscoring the importance of optimized inlet design and plasma generation under rarefied atmospheric conditions.

The most promising path for ABEP acceleration mechanisms is a hybrid approach that combines the controllability of electrostatic acceleration with the scalability and robustness of electromagnetic and wave-based methods. Such hybrid architectures trend towards improved efficiency, increased operational lifetime, and broader mission applicability. At present, most demonstrated VLEO propulsion systems operating above approximately 160 km rely on atomic oxygen breathing. To fully exploit the air-breathing propulsion concept, future missions will likely need to operate at lower altitudes, where higher atmospheric density and more favourable composition enable sustained, long-duration propulsion.

## Data Availability

All data generated or analysed during this study are included in this published article.
